# Autophagy in Sensorineural Hearing Loss: Jekyll or Hyde?

**DOI:** 10.3390/ijms27042053

**Published:** 2026-02-22

**Authors:** María Beatriz Durán Alonso

**Affiliations:** 1 Department of Biochemistry and Molecular Biology and Physiology, Faculty of Medicine, University of Valladolid, 47005 Valladolid, Spain; mariabeatriz.duran@uva.es; 2 Unit of Excellence, Institute of Biomedicine and Molecular Genetics (IBGM), University of Valladolid-CSIC, 47003 Valladolid, Spain

**Keywords:** autophagy, mitophagy, aging, ototoxicity, hearing loss

## Abstract

Autophagy plays a key role in the development and homeostasis of the cochlear organ. Alterations in the autophagic pathways have been associated with damage to auditory cell types and hearing impairment caused by an array of factors like age, ototoxicity, exposure to high levels of noise, or genetic mutations. Cochlear damage frequently entails mitochondrial dysfunction, impaired mitophagy and the accumulation of high concentrations of free radicals. This review summarizes the observations made to date on the autophagic function in response to cochlear damage and the results of either activating or inhibiting these processes. The data demonstrate that autophagic activity is cell context-dependent and varies according to the cochlear cell type, the toxic agent, its levels and the length and timing of its administration; other factors that influence the autophagic response may be external to the auditory system or related to epigenetic changes or the expression of genetic variants. Modulation of the autophagic status has an effect on auditory cell loss and the progression to hearing impairment and this approach has thus become a promising avenue towards the protection of the hearing function. Nonetheless, this is no easy task and it will require the identification of reliable biomarkers to evaluate the dynamics of autophagic activity as well as the development of specific autophagy modulators that do not exert toxicity.

## 1. Introduction

The mammalian cochlea is the result of a perfect integration of mechanisms that control growth and patterning [1], which gives rise to a highly organized array of sensory mechanoreceptors, supporting cells and sensory neurons; these cells become post-mitotic during embryogenesis, generally preceding the final stages of differentiation. Some of these cell types, like the hair cells (HCs) and the spiral ganglion neurons (SGNs) exhibit high susceptibility to injury by a whole range of stimuli such as oxidative stress, age, drugs and noise. Also, it appears that they could be enriched in so-called long-lived proteins (LLPs) that persist for very long times in the cell, instead of undergoing frequent turnover [2]. Since these are post-mitotic cells, they cannot dilute misfolded or damaged proteins through proliferation cycles, which makes the regulation of proteostasis especially relevant. The accumulation of aberrant proteins frequently leads to the production of free radicals and reactive oxygen species (ROS) and, ultimately, to cell death; in these instances, autophagy, a catabolic process that supplies energy to the cell by degrading cytoplasmic waste products [3,4], plays a key role in determining cell fate.

There are different types of autophagy: (a) microautophagy, whereby damaged organelles are engulfed and directly degraded by lysosomes, (b) chaperone-mediated autophagy, where the cargo is marked by the chaperone peptide HSC70 (heat shock cognate protein 70) and translocated to lysosomes or endosomes for its degradation, and (c) macroautophagy, which I will hereafter refer to as autophagy, where the proteins to be discarded from the cell are identified via adaptors such as the sequestome 1 (SQSTM1/P62) protein prior to their sequestration into autophagosomes. Additional forms of autophagy have been described, like mitophagy, for the disposal of dysfunctional mitochondria, preventing its accumulation, and pexophagy, for the degradation of damaged or excess peroxisomes [5].

Autophagy [6] commences with the formation of a phagophore that expands to give rise to a double-membraned autophagosome. The autophagosome engulfs aberrant proteins and damaged organelles and fuses to a lysosome, forming an autolysosome. Subsequently, the contents in the autolysosome are degraded by lysosomal acid hydrolases. The process is regulated by AMPK (adenosine monophosphate-activated protein kinase) and generally conveys the inhibition of the mTOR (mammalian target of rapamycin) pathway [6,7]. A large number of autophagy-related (ATG) proteins participate at different stages in the process. Beclin 1 binds to the phosphatidylinositol 3-kinase complex, leading to the activation and cleavage of LC3 (microtubule-associated protein light chain 3), to generate LC3-I; cytosolic LC3-I is then lipidated, giving rise to LC3-II, which translocates to the autophagosome membrane, promoting autophagosome assembly and cargo recruitment. Once the autophagosome fuses to the lysosome and its contents are degraded, lysosomal permeases release the breakdown products into the cytosol, so that they become available to synthetic and metabolic pathways.

**Figure 1:**
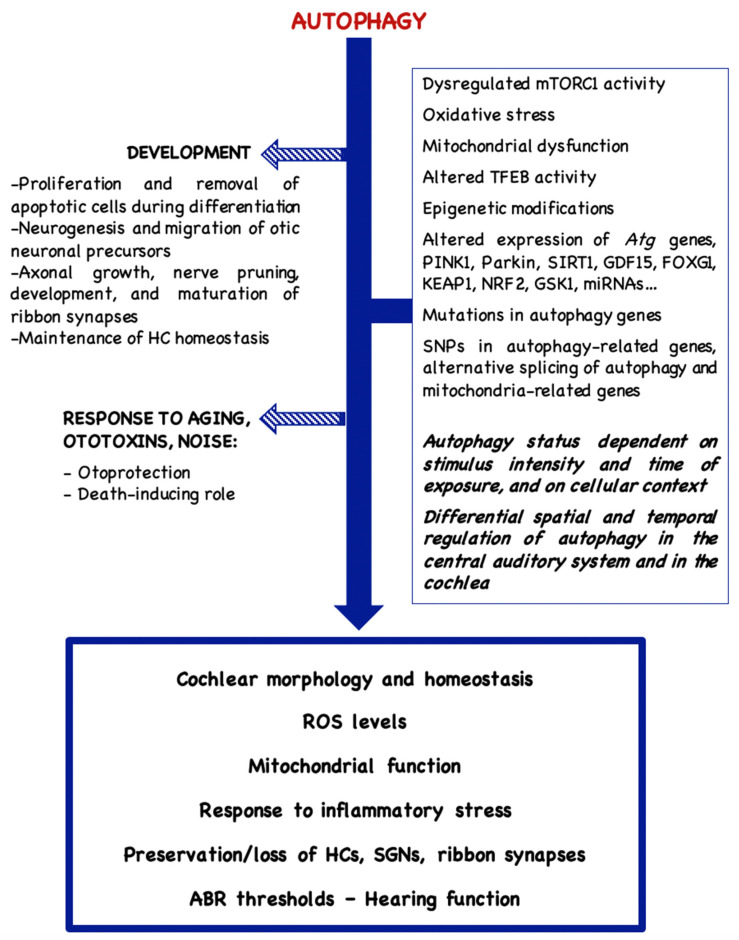
Regulation and roles of autophagy in the auditory organ.

In the auditory organ, autophagy serves cell-specific functions and it is key to maintaining cellular homeostasis and energy supply [8]. It plays a role during development [9,10], it serves a housekeeping role in the mature organ under basal conditions, and it is also activated under stress conditions, such as oxidative stress, nutrient deprivation, inflammation and ischemia [11]. Nonetheless, although autophagy often plays a protective role, the activation of the autophagic process may in other instances lead to programmed cell death, depending on the cellular context and the physiological conditions [8,12,13,14,15,16]. Factors that may modulate the autophagic flux and the various roles played by autophagy in the auditory organ are summarized in Figure 1.

## 2. Autophagy and Development

Autophagy plays a key role in auditory development and maturation. In the mouse cochlea, it becomes up-regulated from perinatal stages to adulthood and key autophagy-related genes such as *Atg*4b, *Atg*5, *Atg*9a and *Beclin*1 peak at postnatal day (P) P30-P60, coinciding with the functional maturation of this tissue [17]. Inhibition of the autophagic flux at the early stages of chicken otic development blocks proliferation, impairs neurogenesis, causes poor axonal outgrowth and leads to aberrant otocyst morphogenesis, with the accumulation of apoptotic cells [18,19]. During this period, autophagy is known to provide the energy that is needed to eliminate the apoptotic cells that result from the differentiation process; it is necessary for the expression of phosphatidylserine on the surface of dying cells so that these may be engulfed by phagocytes and thus its inhibition gives rise to an increase in the numbers of TUNEL-positive cells in the developing otocyst [19]. Homozygous knock-out mice for the *Atg*5 gene do not present autophagosome formation and exhibit progressive HC degeneration from P5 onwards; they are hearing-impaired and already exhibit increased auditory brainstem responses (ABRs) at postnatal weeks 4–8 [20].

Autophagy plays a key role in the migration of otic neuronal precursors from the neurogenic zone and in their differentiation, which affect the formation of the acoustic-vestibular ganglion and the outgrowth of neuronal processes [18]. Interestingly, the application of an ATP source rescues the developmental alterations caused by the inhibition of autophagy. Inhibition of autophagy in organotypic cultures of chicken otic vesicles has confirmed the importance of autophagy in these developmental processes [21]. Autophagic activity increases during SGN development [22], when preferential expression of the autophagy marker LC3B is observed, pointing at the relevance of autophagy during this process [23]. Apoptotic activity peaks at P7 during the postnatal development of rat SGNs and markedly declines at P10-P14, while the expression of LC3-II and Beclin 1 is up-regulated, with maximal expression at P10 and P7, respectively; the levels of P62, which is degraded during the autophagic process, are down-regulated from P1 to P14 [22]. Data obtained by Guo and co-workers (2022) [24] support that robust autophagic activity regulates nerve pruning and the development and maturation of cochlear ribbon synapses between inner HCs (IHCs) and SGNs during auditory development. Ribbon synapses are immature at birth and require morphological and functional development to achieve auditory maturation. In mice, there is a high level of expression of LC3B in the cochleae of P1–P15 animals, which decreases at P28–P30. Autophagy inhibition at P7 with 3-methyladenine (3-MA), which blocks the early stages in the autophagic process, leads to significant elevations in the hearing thresholds from P15 to P30. The recorded hearing deficits appear to be associated with an abnormal morphology of ribbon synapses and reduced IHC exocytosis function in early postnatal mice [25].

Work carried out by Varela-Nieto’s group [26] on the HEI-OC1 (House Ear Institute Organ of Corti 1) cell line [27,28] has unveiled a key role for insulin growth factor 1 (IGF-1) in the development and maintenance of the auditory system. IGF-1 stimulation of HEI-OC1 cultures during their early differentiation promotes their survival. Interestingly, while the application of IGF-1 to undifferentiated HEI-OC1 cultures leads to a progressive increase in the levels of phosphorylated mTOR and to a reduction in the autophagic flux, differentiating cells exhibit marked autophagy induction that cannot be suppressed by IGF-1, with augmented numbers of autophagic vesicles and autophagosomes [26].

## 3. Mitochondrial Dysfunction, Oxidative Stress and Autophagy

Correct mitochondrial function depends on the balance between the generation of newly synthesized mitochondria (mitochondrial biogenesis) and the removal of dysfunctional mitochondria (mitophagy). Mitophagy [29] begins when damaged mitochondria undergo membrane depolarization, leading to the accumulation of the serine/threonine-protein kinase PINK1 (phosphatase and tensin homolog-induced kinase 1) on the outer mitochondrial membrane (OMM). PINK1 then undergoes autophosphorylation and activates the E3 ubiquitin ligase Parkin, which attaches ubiquitin molecules to the damaged mitochondria, targeting them for degradation. The ubiquitin-tagged mitochondria are engulfed by autophagosomes and degraded in autolysosomes. The degradation of damaged mitochondria can also be orchestrated through a PINK1/Parkin-independent pathway, whereby dysfunctional mitochondria are recognized by other factors.

Hearing loss (HL) is frequently associated with impairments in autophagy and/or mitophagy and with the accumulation of oxidative free radicals [29]; ROS overproduction may lead to lower mitochondrial membrane potential (MMP), lipid peroxidation, pro-inflammatory cytokine secretion and senescence [30]. Oxidative stress has been recorded in age-related, noise-induced and ototoxin-induced HL.

Contradictory data have been published on the effect of autophagy on ROS accumulation and cell survival. Autophagy induction may be a protective response of the cell against oxidative stress [31,32,33], as reported by Hayashi and co-workers (2015) [34], who registered autophagy induction in HEI-OC1 cells following treatment with H_2_O_2_. Application to the cultures of the autophagy inducer rapamycin ameliorated cell loss, while *Atg*7 knock-down induced an increase in cell death by necrosis; this was due to the inactivation of the cytoprotective KEAP1 (Kelch-like ECH-associated protein 1)/NRF2 (nucleus factor erythroid 2-related factor 2) signaling pathway, which activates the expression of cytoprotective genes in response to oxidative stress [34]. A pro-survival effect of autophagy induction was also observed by Lim and colleagues (2025) [31] when applying the natural compound mangiferin to H_2_O_2_-treated HEI-OC1 cultures. The compound induced autophagic activity and reduced ROS levels, compared to H_2_O_2_-only treated cultures. In a guinea pig model treated with kanamycin, mangiferin helped preserve HC numbers and protected ribbon synapses from damage, yielding improved ABR thresholds [31]. Protection of H_2_O_2_-treated HEI-OC1 cultures against oxidative stress was also achieved through the ubiquitination of the voltage-dependent anion channel 1 (VDAC1), localized on the OMM. VDAC1 is a substrate for Parkin and its ubiquitination regulates mitochondrial function and mitophagy by promoting autophagosome formation and lysosomal binding to the mitochondria labeled for selective degradation. This mechanism leads to a decrease in ROS production and supports cell survival [35,36].

Sustained oxidative stress induced by glucose oxidase/glucose decreases the viability of marginal cells of the stria vascularis and increases intracellular ROS. Jiang and co-workers (2018) [37] recorded the stimulation of parthanatos and autophagy in rat cochlear marginal cells via the activation of poly(ADP-ribose) polymerase-1 (PARP-1) [37]. Parthanatos is a modality of cell death that conveys the hyper-activation of PARP-1 and excessive polyADP-ribose (PAR) polymer synthesis. PAR then translocates from the nucleus to mitochondria, binding to apoptosis-inducing factor (AIF) and triggering its translocation to the nucleus, inducing nuclear condensation and ensuing cell death; alternatively, abnormal accumulation of PAR polymer may cause cell death by inducing mitochondrial depolarization. PARP-1-induced autophagy is a cytoprotective mechanism and its inhibition exacerbates parthanatos, thus indicating a pro-survival role of autophagy against oxidative stress in the marginal cells. Jiang and colleagues (2018) [37] hypothesized that mild levels of ROS could be triggering the mild activation of PARP-1, which induced pro-survival autophagy and DNA repair, while higher levels of oxidative stress could be leading to PARP-1 overactivation, ATP depletion and altogether to cellular energy failure and parthanatos.

On the other hand, Wu and co-workers (2020) [38] described the protective effect of 2,3,4′,5-tetrahydroxystilbene-2-O-β-D-glucoside (THSG) on otic UB/OC-2 cells treated with H_2_O_2_. THSG exhibited free radical scavenger activity like that of ascorbic acid. It reverted the loss of mitochondrial membrane potential (MMP), inhibiting the mitochondrial-dependent apoptosis pathway; it also enhanced the expression of NRF2, its dissociation from KEAP1 and its translocation into the nucleus, with the concomitant increase in the expression of antioxidant/detoxifying proteins such as heme oxygenase-1 (HO-1) and NAD(P)H:quinone oxidoreductase-1 (NQO-1). Of note, not only did THSG protect the UB/OC-2 cells from oxidative stress by inhibiting apoptosis, but it also inhibited H_2_O_2_-induced autophagy, reverting the H_2_O_2_-induced increase in LC3-II protein levels.

## 4. Autophagy and Age-Related Hearing Loss

Self-repair mechanisms decline during aging, with the concomitant accumulation of abnormal or damaged molecules and organelles [23]. Hallmarks of aging are impaired protein homeostasis, decreased activities of the autophagy-lysosomal and the ubiquitin-proteasome systems, and mitochondrial dysfunction, with excessive ROS production [39,40].

The incidence of HL among individuals from 60 to 80 years old ranges from 21% to 27% [41,42]. Increased oxidative stress has been shown in the aging cochlea [43] and it is known that autophagy becomes activated as a response to try to limit ROS-induced tissue damage [44,45]. Increased levels of autophagy-associated proteins and LC3-positive autophagosomes have been observed in the cochlea of aged animals [16,46], which is considered a means to maintain hearing function, delaying the onset of age-related hearing loss (ARHL). However, a rise in the levels of P62 is also observed in many instances, indicating that the autophagic process is not proceeding normally but that it is blocked [16] and contributes to increased cellular damage. As a result, aging is accompanied by a senescent phenotype of the HCs and, frequently, by a reduction in autophagic activity, with decreased LC3-II and increased P62 protein levels and attenuated transcription of *Atg* genes; aging is also associated with reduced nuclear localization of the autophagy master regulator TFEB (transcription factor EB), which enters the nucleus upon activation and up-regulates the expression of autophagy-related genes [47]. RNAseq transcriptomic analyses of inner ear cell populations isolated from adult and aged CBA/J mice have also pointed at a possible epigenetic silencing of HC genes during aging and a down-regulation of autophagy and mTOR signaling pathways [48]. Very interestingly, a study conducted by Peng and collaborators (2022) [49] has shown that the set of autophagy genes that are differentially expressed between normal-hearing mice and aging mice with HL differ depending on the degree of HL (i.e., mild vs. severe HL), indicating the involvement of different molecular mechanisms in presbycusis. 

Naturally aged C57BL/6J mice present oxidative stress and increased SGN apoptosis, with increased mTOR activity and decreased expression of autophagy-related genes and autophagosomes [50]. Rapamycin administration reduces mTOR activity, increases autophagy and improves ABR thresholds, reducing the rate of SGN apoptosis and enhancing SGN density [50]. Additionally, a decrease in the levels of SIRT1 (sirtuin 1) and GDF15 (growth differentiation factor 15) has been reported in ARHL rats, accompanied by elevated apoptosis and blocked autophagy [16]. SIRT1 is an NAD-dependent histone deacetylase known to induce autophagy/mitophagy, putatively via ATG9A deacetylation [51,52,53]. GDF15 overexpression restores autophagic flux and reduces ABR thresholds and HC loss, via SIRT1 up-regulation. Changes in the expression of the nuclear transcriptional regulator FOXG1 (Forkhead box G1) [54] have also been described in a D-galactose-induced aging model. FOXG1 inhibited ROS accumulation through autophagy activation and preserved HC function and survival [55]. Increasing D-galactose concentrations led to a decrease in FOXG1 expression, while aspirin administration resulted in increased FOXG1 levels and promoted its protective activities against ARHL.

Various bioactive compounds have been shown to promote cell survival in aging models through the induction of autophagy. The curcumin analog C1, a stronger autophagy activator than curcumin, exerted an antioxidative effect [56] and reduced HC loss in aged C57BL/6 mice and in cultures of aging cochlear explants. C1 reverted the aging-associated inhibition of the translocation of TFEB to the nucleus [47,57], alleviated senescence and delayed ARHL progression, reducing ABR threshold shifts in aging mice. Knock-down of TFEB or treatment with the autophagy inhibitor chloroquine, which blocks autolysosome formation, eliminated the protection conferred to the HCs by C1, demonstrating that its protective action is linked to the autophagic process [47,56]. Another study by Salam and co-workers (2021) [58] described the protective effects of thymoquinone, which preserved HC morphology and enhanced SGN survival, improving auditory function in aging C57BL/6J mice and delaying the onset of ARHL. This potent antioxidant down-regulated the expression of the pro-apoptotic protein BAK1 (Bcl-2 antagonist/killer 1) and up-regulated the expression of SIRT1. In agreement with these results, Xiong and co-workers (2019) [59] reported the enhanced expression of PINK1 and Parkin in the HCs of aged mice, evidencing a selective activation of mitophagy, and a delay in the progression of ARHL following SIRT1 overexpression. Very interestingly, Jin and colleagues (2025) [35] reported the decreased expression of PINK1 and Parkin in the central auditory system with advancing age. These observations would tie in with the observations by He et al. (2021) [55] of different spatial and temporal regulation of autophagy in the central auditory system and in the cochlea; this group ascribed the differences in autophagy levels to the expression levels of FOXG1.

On the other hand, excessive autophagy has been implicated in ARHL pathogenesis [60]. Work on the senescence-accelerated mouse prone 8 (SAMP8) mutant mice that present ARHL has unveiled autophagic stress in these animals [61]. Work conducted by Menardo and co-workers (2012) [61] supported an initial pro-survival function of the autophagic process that is activated in these mice; on the contrary, the excessive activation of the pathway in older animals was associated with the accumulation of damaged mitochondria and protein aggregates in SGNs and with cell death. Guan et al. (2025) [62] reported the up-regulation of HDAC11 (histone deacetylase 11) in the HCs of aging mice. HDAC11 inhibited the expression of PINK1/Parkin proteins, leading to impaired mitophagy and mitochondrial dysfunction and to the attenuation of ARHL in older mice, arguing for the inhibition of mitophagy as a means to improve the hearing function. Contrarily, Lin and co-workers (2019) [63] reported that overexpression of the cytosolic GTPase DRP1 (Dynamin-related protein 1) rescued mitochondrial function in senescent HEI-OC1 by initiating mitophagy and that DRP1 inhibition in aged mice inhibited mitophagy and exacerbated ARHL.

Additional studies by Fu and co-workers (2018) [64] have demonstrated that mTORC1 activity becomes dysregulated during aging, contributing to ARHL. Inhibition of mTORC1 signaling in mice by conditionally ablating the expression of the mTORC1-specific component Raptor in the neurosensory epithelium promoted HC survival and preserved the numbers of synaptic ribbons in aged animals, alleviating ARHL [64,65]. On the contrary, constitutive mTORC1 activation through deletion of the *Tsc*1 (tuberous sclerosis complex 1) gene, which is part of the TSC1/TSC2 complex that tonically inhibits mTORC1, led to autophagy inhibition and accelerated HC loss and early-onset progressive HL; this was due to ROS-induced cell damage causing premature senescence and could be prevented by treatment with either the antioxidant N-acetylcysteine or with the mTORC1 inhibitor rapamycin; Fu and co-workers (2018) [64] identified the peroxisome as the source for ROS and autophagy disruption.

There is a correlation between autophagy impairment and cell senescence. Sodium arsenite causes HL that resembles ARHL by promoting ROS generation and premature auditory senescence, likely through the inactivation of TFEB and blockade of the autophagosome–lysosome fusion. The proportion of senescence-associated β-galactosidase-positive cells was reduced by inhibiting mTOR, which induced lysosomal activation and autophagosome–lysosome fusion and promoted autophagy [66]. Wei and colleagues (2025) [67] demonstrated that the application of low D-galactose concentrations induced autophagy in HC cultures, while higher concentrations of D-galactose disrupted normal cellular autophagy and induced mitochondrial damage and HC senescence; this was ameliorated by the overexpression of the zinc-finger protein RONIN, a shuttle protein that translocates into the nucleus and leads to increased autophagic and lysosomal activity. RONIN, which decreased following D-galactose treatment, was shown to interact with host cell factor C1 (HCFC1), enhancing the expression and the transcriptional activity of TFEB and inducing autophagy, as indicated by a reduction in the levels of senescence-related proteins P16, P21 and g-H2A.X and increased LC3B-II, cathepsin B and cathepsin D levels. The promotion of autophagy by RONIN overexpression led to enhanced HC survival. In another series of experiments, the application of the neurotrophic factor neuregulin to cochlear organotypic murine cultures treated with D-galactose resulted in attenuated senescence, with lower ROS levels and P21 protein expression in the cultures. Neuregulin was mainly expressed by cochlear supporting cells and its conditional overexpression in these cells protected against HC and SGN loss caused by aging, maintaining hearing thresholds [68]. These beneficial effects were all associated with increased autophagic flux and down-regulated caspase-3 activity, induced by neuregulin.

Senescence of HEI-OC1 cells and aging in C57BL/6J mice have also been associated with excessive autophagy activation and to an up-regulation of the DLK/JNK (dual leucine zipper kinase/c-Jun N-terminal kinase) pathway [69]; inhibition of autophagy in the cell line and of DLK or JNK3 in the mice led to reduced senescence and improved hearing. Also, work by Lv and co-workers (2022) [46] correlated senescence in H_2_O_2_-treated HEI-OC1 cells with autophagic activity. Senescent cells down-regulated PIN1 (prolyl-isomerase), the regulator of post-phosphorylation; *in vivo* experiments demonstrated that PIN1 inhibition in the HCs of young mice promoted autophagy and led to increased ABR thresholds in these animals.

Aging has been associated with a greater sensitivity to inflammatory stress. In this context, He and co-workers (2019) [70] reported decreased FOXG1 levels in aged OC-1 cells and in aged cochlear explants. FOXG1 behaved as an autophagy regulator that exerted a protective role against inflammatory injury, although extensive cell damage ultimately led to reduced FOXG1 expression and increased apoptotic rates [70]. FOXG1 levels were further decreased when aged OC-1 cultures were exposed to pro-inflammatory conditions, associated with an enhanced susceptibility to inflammatory damage and parallel to a further reduction in autophagic activity. Aspirin activated FOXG1 expression and reverted these effects, promoting the survival of mimetic aging OC-1 cells [70].

Various miRNAs have been associated with autophagy dysregulation during aging [60,71,72]; Zhang and colleagues (2024) [72] demonstrated increased levels of miR-130b-3p in the cochleae of aged C57BL/6 mice that exhibited higher ABR thresholds than control mice, together with decreased levels of the autophagy regulator PPARγ. A reduction in PPARγ expression was also confirmed in HEI-OC1 cells transfected with a miR-130b-3p agomir, together with a loss of cell viability. Down-regulation of this miRNA led to an increase in the levels of the autophagy biomarkers ATG5, Beclin 1 and LC3B II/I [72]. Up-regulation of cochlear miR-34a was also correlated with ARHL in C57BL/6 mice and cochlear HC loss and death in HEI-OC1 cultures, together with an impairment of the autophagic flux [71]; these effects correlated with a down-regulation of the ATG9A protein, which promotes the nuclear translocation of TFEB [73]. Treatment with rapamycin restored TFEB’s nuclear localization and autophagic activity in aging HCs, preserved HC numbers and delayed ARHL in C57BL/6 mice [73]. Suppression of miR-34a expression in HEI-OC1 cells with ursodeoxycholic acid also restored autophagy and ATG9A expression and protected the cells from dying. In agreement with these results, Xiong and co-workers (2019) [59] demonstrated a protective effect of down-regulating miR-34a on H_2_O_2_-treated HEI-OC1 cultures, through the activation of mitochondrial autophagy.

Cholesterol accumulation is a risk factor for ARHL [74] and leads to increased mTORC1 signaling and reduced levels of TFEB. Treatment of an ARHL mouse model with atorvastatin, a cholesterol synthesis inhibitor, reduced the cholesterol levels and preserved lysosomal function and autophagy, via mTORC1 inhibition and TFEB activation, and resulted in delayed HL [74]. Similar results were obtained with a stress-induced premature senescence HEI-OC1 model. Table 1 summarizes the data obtained from aging models that correlate autophagy status with cell and hearing loss.

## 5. Autophagy and Ototoxin-Induced Hearing Loss

Over 150 ototoxic compounds have been described that may cause damage to cells in the inner ear [75]. Amongst these, there are platinum-based compounds (e.g., cisplatin [76]), aminoglycoside antibiotics (AGs) [77], and certain environmental pollutants [78].

### 5.1. Aminoglycoside Antibiotics

A percentage of 20–47% of patients treated with AGs have been reported to suffer from HL [79]. AGs are taken up by cochlear HCs and SGNs and induce ROS accumulation, apoptosis and cell loss *in vivo*. AG entry into the HCs induces the translocation of RIPOR2 (RHO family interacting cell polarization regulator 2), required for stereocilia morphogenesis, from the base of stereocilia to the pericuticular area. It then recruits and activates GABARAP (gamma-aminobutyric acid receptor-associated protein) and other autophagy-related proteins, leading to autophagy dysregulation, HC death and HL [80]. Reduced expression of RIPOR2 or GABARAP, or of other autophagy-associated proteins, conferred resistance to AG-mediated ototoxicity, indicating that RIPOR2-induced autophagy plays a key role in AG-induced HL [80,81,82].

Gentamicin (GM) induced mTORC1 hyperactivation and yielded decreased densities of SGNs and neurite outgrowth in cultured SGN explants [83]; rapamycin exerted a protective effect against GM, reverting its effects. Application of osthole, a plant-derived coumarin, to GM-treated HEI-OC1 cells and zebrafish larvae attenuated GM-induced oxidative stress, reducing HC apoptotic rates; the compound inhibited lipid peroxidation and restored autophagy [84]. Although GM treatment of cochlear explants and the HEI-OC1 cell line caused delayed ototoxicity associated with a time-dependent increase in the autophagosome markers LC3-II and Beclin 1, it also induced a decrease in the levels of RAB7, which plays an important role in the fusion of autophagosomes with lysosomes [85]. Thus, GM treatment was followed by autophagosome accumulation, due to defects in the late endosome–lysosome stages of autophagy, resulting in cell death. Co-treatment with GM and either 3-MA or chloroquine led to increased cell death rates; *Atg*5 knock-down also decreased cell viability. On the contrary, co-treatment with GM and rapamycin enhanced autophagic flux and prevented GM-induced cell death [85], indicating that AG-induced cell death may result from autophagic dysfunction. GM was also shown to aggravate mitochondrial dysfunction in cybrids carrying a m.1494C>T mutation in mitochondrial rRNA [86]. This type of mutation renders mitochondrial ribosomes more similar to bacteria and therefore more susceptible to AG binding. Mitochondria became elongated and fused, forming numerous networks to optimize mitochondrial function; mitophagy was strongly stimulated to eliminate damaged mitochondria and protect the cells from apoptosis [86]. On the other hand, Ma and co-workers (2017) [87] registered increased LC3-II levels and autophagy induction following GM treatment of murine cochlear cultures. In this case, autophagy inhibition by 3-MA ameliorated GM-induced HC loss, as 3-MA interfered with the pro-apoptotic function of ATG12 [87].

Li and colleagues (2022) [88] reported the induction of autophagy in neomycin-treated HEI-OC1 cultures. Neomycin increased the levels of autophagy-related nuclear dot protein 52 (NDP52) by inhibiting the expression of its negative regulator, miR-489. The antioxidant fasudil inhibited apoptosis in the cultures by inhibiting neomycin-induced ROS production and mitophagy activation, protecting the cells from apoptosis. In contrast, Liu et al. (2024) [89] attenuated HC loss induced by neomycin treatment of HEI-OC1 cells and cochlear explants by applying exosomes from umbilical cord-derived mesenchymal stem cells that activated autophagy. Increased autophagic activity decreased mitochondrial oxidative stress and promoted HC survival, reducing the proportion of apoptotic cells [89]. Exosome application to the cultures increased the levels of the autophagy-associated proteins LC3-II and Beclin 1, reduced those of SQSTM1/P62, and promoted autophagosome–lysosome fusion. Importantly, autophagy inhibition by 3-MA treatment or *Atg*5 knock-down reduced the beneficial effects exerted by the exosomes, pointing at autophagy as an essential process for exosome-mediated protection of HEI-OC1 cells and cochlear explants [89]. In addition, an up-regulation of endocytic genes was registered in HCs treated with exosomes; administration of endocytic inhibitors prevented the activation of autophagy by the exosomes, indicating that exosomes must promote endocytosis to activate autophagy.

Autophagy induction through the inhibition of the phosphodiesterase PDE4 in HEI-OC1 cultures and in mouse models treated with kanamycin preserved cell viability, IHC ribbon synapses and hearing function [90]. Autophagosome formation, suppressed by kanamycin treatment, was restored following PDE4 inhibition; in turn, blockade of the autophagic flux by either treating the HEI-OC1 cultures with chloroquine or by down-regulating the expression of the *Atg*7 gene inhibited the protective effect of inhibiting PDE4. In addition, kanamycin treatment induced SGN degeneration, with the presence of TFEB in the cytosol; the mTOR inhibitor temsirolimus promoted TFEB translocation to the nucleus, restored autophagy and attenuated SGN degeneration [91,92].

### 5.2. Cisplatin

Cisplatin is a highly ototoxic agent that causes OHC loss, leaving scattered numbers of surviving IHCs. ROS accumulation is considered the primary mechanism by which cisplatin induces apoptosis in inner ear cells [93]; excessive ROS production can promote the inflammatory response, contributing to apoptosis. Xu and co-workers (2018) [94] described increased numbers of TUNEL-positive cells and HL in C57BL/6 mice treated with cisplatin; the levels of both apoptotic (BAX, cleaved caspase-3) and autophagic (Beclin1, LC3-II) markers were increased, indicating the promotion of both the apoptosis and the autophagy pathways. Conflicting results have been reported for the role of autophagy in cisplatin-induced ototoxicity and it seems that autophagy may have a protective or a pro-apoptotic role, depending on the timing and severity of cellular damage [93].

Liu and co-workers (2021) [95] registered increased autophagy in cultures of cochlear SGNs following cisplatin treatment, with enhanced autophagosome synthesis and fusion of autophagosome–lysosome complexes. Further activation of the autophagic flux with rapamycin reduced ROS accumulation and attenuated SGN apoptosis and HL. Cisplatin also enhanced the expression of the antioxidant enzyme peroxiredoxin 1 (PRDX1), which activates autophagy; PRDX1 deficiency in Prdx1^−/−^ mice resulted in increased SGN loss following cisplatin treatment [95]. Very interestingly, although PRDX1 is also expressed in cochlear HCs, its expression levels did not change in these cells following cisplatin treatment and no difference was recorded in the extent of HC loss between wild-type mice and Prdx1^−/−^ animals. Cisplatin treatment also caused a dose-dependent increase in YTHDF1 expression. This is an m6A (N6-methyladenosine)-modified mRNA-binding protein expressed in the cytoplasm of HEI-OC1 cells and of murine cochlear HCs and its expression decreases gradually from P0 to P30. YTHDF1 exerted a protective role by promoting the translation of ATG14 and enhanced autophagy, alleviating cisplatin-induced toxicity [96]. Cisplatin treatment of *Ythdf*1^−/−^ C57BL/6 mice resulted in more severe HC damage than that seen in wild-type animals. Additionally, ROS generation following cisplatin treatment led to the activation of the PINK1/Parkin pathway in HEI-OC1 cells, cochlear HCs and SGNs. PINK1 protected the cells from cisplatin by promoting autophagy, clearing damaged organelles and inhibiting the p-JNK apoptotic pathway. While addition of the ROS scavenger N-acetyl-L-cysteine ameliorated autophagic activity and reduced JNK pathway apoptosis, silencing of PINK1 expression also decreased autophagy but led to more severe cisplatin-induced apoptosis [97].

Interestingly, it has been shown that the effects of cisplatin treatment on gene expression may vary depending on drug concentrations. Mu and co-workers (2023) [98] registered an increase in FOXG1 expression and in autophagic activity in CBA/CaJ mice and HEI-OC1 cultures that had been treated with low doses of cisplatin; on the other hand, they observed the opposite effect when higher concentrations of the drug were applied. In the latter case, an increase in the levels of the epigenetic post-translational modification H3K9me2 (Histone H3 lysine 9 dimethylation) was observed, together with a reduction in FOXG1 expression and autophagic flux, ROS accumulation and HC death [98]. Mu and colleagues (2023) [98] concluded that H3K9me2 may regulate autophagy through a FOXG1-dependent pathway that is dependent on the expression of the microRNAs miR-34a, miR-96, miR-182 and miR-183, as their down-regulation following cisplatin treatment was directly correlated to the reduction in autophagic activity. Inhibition of these miRNAs also correlated with increased apoptotic rates. *In vivo* work showed that inhibition of H3K9me2 in cisplatin-treated CBA/CaJ mice could protect them against cisplatin-induced HL [98]. On the other hand, Wang and colleagues (2023) [99] recorded an increase in the expression of miR-34a in C57BL/6 mice and HEI-OC1 cultures following cisplatin administration; this was accompanied by suppression of DRP1 (Dynein-related protein 1) levels, mitophagy inhibition and increased cell damage. DRP-1 is a downstream target of miR-34a and regulates mitochondrial homeostasis. A function for miR-34a in regulating mitochondrial function in the response to cisplatin treatment was supported by the results obtained following miR-34a inhibition, showing increased DRP1 expression, reduced ROS levels, improved mitochondrial function and attenuated cisplatin-induced ototoxicity [99].

Fang and Xiao (2014) [32] demonstrated attenuation of cisplatin-induced HL in rats following autophagy activation by rapamycin. Reduced OHC loss, decreased ABR shifts and a decrement in the levels of the oxidative marker malondialdehyde were observed in these animals, compared to cisplatin-only treated rats. Similar results were obtained by Xu and co-workers (2025) [33] on mouse cochlear explants; rapamycin was shown to counteract cisplatin-induced activation of mTORC1 leading to ROS accumulation and cell death. Other autophagy inducers have been shown to provide protection against cisplatin-induced HC loss and HL. The natural disaccharide (α,α-1,1-glucoside) trehalose ameliorated cisplatin-induced oxidative stress and mitochondrial dysfunction; it activated autophagy by increasing *Tfeb* mRNA expression and promoting its translocation to the nucleus and reduced HC loss in mouse cochlear explants and in HEI-OC1 [100]. Application of 3-MA or transfection with *Tfeb*-siRNA abolished the protective action of trehalose on cisplatin-treated cultures. Application of the calcineurin inhibitor cyclosporin A also abolished the effect of trehalose; it prevented TFEB nuclear translocation, inhibiting autophagy, and promoted cisplatin-induced apoptosis [100].

In another series of experiments, application of metformin to cisplatin-treated HEI-OC1 cultures induced autophagy and increased cell survival rates [101]. Co-administration of cisplatin with metformin in zebrafish demonstrated a protective effect of metformin against cisplatin-induced HC loss that did not seem to involve HC regeneration. The same otoprotective effect was recorded in a mouse model, where metformin extended OHC survival and protected the animals against cisplatin-induced HL. The reported findings indicated that metformin alleviated cisplatin-induced ototoxicity, possibly through AMPK/FOXO3a-mediated autophagy, whereby the AMPK target FOXO3a up-regulated the transcription of autophagy-related genes. Data obtained by Lai and co-workers (2025) [102] also support the involvement of AMPK in the protective action of metformin, since AMPK inhibition abolished the ability of metformin to trigger mitophagy and prevent mitochondrial dysfunction and apoptosis caused by the oxidative stress inducer tert-butyl hydroperoxide in HEI-OC1 cultures.

Zhang and co-workers (2024) [103] identified a chiisanoside derivative that up-regulated the expression of the *Lrp*6 (low-density lipoprotein receptor-related protein 6) gene and inhibited GSK3β in HEI-OC1 cells and in murine cochlear HCs treated with cisplatin. GSK3β is a serine/threonine kinase that negatively regulates autophagy and its inhibition promotes the translocation of TFEB to the nucleus, the activation of autophagy and the inhibition of ROS generation [104]. Nobiletin, a citrus polymethoxy flavonoid, attenuated cisplatin-induced apoptosis and oxidative stress in HEI-OC1 cells and C57BL/6 cochlear explants through the activation of autophagy [105], facilitating the nuclear translocation of NRF2 and the transcription of its target genes.

On the other hand, a cell death-promoting effect of autophagy was observed in cisplatin-treated HEI-OC1 auditory cells, following an initial cytoprotective effect [106]; hydroxychloroquine pre-treatment to inhibit autophagy protected cells from cisplatin ototoxicity. Application to cisplatin-treated HEI-OC1 cultures of the ethyl ester form of meclofenamic acid MA2, an anti-inflammatory drug, also inhibited an excessive activation of autophagy by cisplatin and reduced oxidative stress and apoptosis [107]. The production of a large amount of ROS, mitochondrial damage, apoptosis and autophagy following exposure of zebrafish and HEI-OC1 cells to cisplatin was attenuated by treatment with lysophosphatidic acid, which protected against HC death while inhibiting a cisplatin-induced increase in autophagic activity [108]. The LPA_1_ receptor participated in this protection since silencing of the receptor in HEI-OC1 cells blocked LPA-mediated protection against cisplatin and promoted apoptosis.

Cisplatin can induce various types of cell death. One of these is ferroptosis [15,93,109], a form of regulated cell death that involves intracellular iron accumulation, with iron-dependent lipid peroxide build-up. Ferroptosis contributes to cisplatin-induced OHC loss and hearing deficits in C57BL/6 mice [15]. Cisplatin treatment was shown to induce lipid peroxidation in C57BL/6 mice and activated ferritinopathy in HEI-OC1 cultures, leading to ferritin-iron release and ferroptosis via autophagy [93]. Therefore, autophagy performed a pro-death function, contributing to the accumulation of intracellular free iron, and its inhibition with chloroquine alleviated cisplatin-induced ferroptosis. Ferroptosis inhibition attenuated cisplatin toxicity in HEI-OC1 cells and cochlear explants and reduced HL *in vivo*. ROS generation and inflammation caused by cisplatin may contribute to HC death through endoplasmic reticulum stress (ERS) and ER autophagy, with the activation of ER autophagy proteins [110]. Knock-down of the ER autophagy receptor FAM134B decreased cisplatin-induced ER autophagy and ototoxicity. Similarly, knock-down of the inflammatory mediator STAT1 (signal transducer and activator of transcription 1), which is activated during cisplatin- and GM-mediated ototoxicity, induced the expression of LC3-II and Beclin 1 and protected HCs from ototoxicity [111].

### 5.3. Other Bioactive Compounds

There are other bioactive compounds that also affect autophagy and cochlear cell survival. One of these is acetaminophen (paracetamol); abuse of analgesics like paracetamol may cause ototoxicity through the induction of oxidative and ER stress. Application of paracetamol to HEI-OC1 cells and cochlear explants caused decreased activity of lysosomal enzymes and impaired autophagy [112]. Down-regulation of autophagy led to elevated ROS and ER stress levels, enhancing apoptosis.

Sevoflurane is one of the most used inhaled anesthetics during labor. However, it has been observed that exposure of mouse offspring to sevoflurane *in utero* can cause hearing impairment [113]. Although sevoflurane did not cause HC damage or reduced HC numbers, it induced a reduction in cochlear ribbon synapses (with partial restoration after sevoflurane withdrawal) and an increase in mitochondrial ROS production, accompanied by decreased autophagy. These alterations resulted in an elevation of the ABR thresholds. Ototoxicity has also been attributed to high doses of caffeine. Caffeine injection in C57BL/6 mice at a concentration of 120 mg/kg/day for 7 and 14 days resulted in a significant increase in auditory threshold shifts, compared to control animals, with HC and SGN loss [114]. Transcriptome analysis identified 1808 differentially regulated genes; the most differentially regulated gene was *Sgk*1, considered to be a modulator of autophagy and apoptosis. *In vitro* work on HEI-OC1 cells demonstrated that caffeine inhibited cell viability by promoting autophagy and apoptosis via the SGK1/HIF-1α pathway. GSK1 inhibition and thus autophagy inhibition protected HEI-OC1 cultures and HCs in cultures of neonatal rat basilar membrane from caffeine-induced damage. Mouse models with elevated levels of uric acid (UA) present higher ABR thresholds than control animals [115]. UA-induced ototoxicity was demonstrated in HEI-OC1 cultures and in HCs in organotypic cochlear cultures, where it activated ferritinophagy; blockage of the autophagic flux with chloroquine ameliorated UA cytotoxicity.

Ototoxicity has also been associated with exposure to some environmental toxins. Cadmium, an environmental and occupational toxin, can induce SGN degeneration and HL [116]. Li and co-workers (2022) [116] reported impairment of the autophagic flux in murine SGN explants exposed to cadmium and recorded a protective action of metformin on the cultures through autophagy restoration. Additionally, lead exposure causes or aggravates hearing impairment. Adult guinea pigs exposed to lead suffered HL and exhibited a gradual reduction in SGN numbers after 60 days; no changes were observed in the numbers or morphology of HCs. The expression of autophagy-related proteins ATG5, ATG6, and LC3B was increased in brainstem after 30 days, pointing at the auditory nerve conduction pathway as the main target and suggesting a possible protective role of autophagy in the early stages of lead exposure [117]. Treatment of HEI-OC1 cells with the environmental/chemical pollutant 2,2′,4,4′-tetrabromodiphenyl ether (BDE-47) induced increased expression of the ligand-activated nuclear transcription factor aryl hydrocarbon receptor (AHR), an environmental stress factor known to affect the autophagic flux in xenobiotic-induced HL. Increased AHR expression resulted in mitochondrial dysfunction and ER stress in the cells, with decreased levels of the antioxidant protein KEAP1, up-regulation of NRF2, HO-1 and NOQ1, and elevated intracellular ROS levels. These changes resulted in an increase in the number of autophagosomes, with augmented LC3B-II and phosphorylated P62 (p-P62) levels indicating autophagy inhibition [78]. *In vivo* work on weaned pigs demonstrated that exposure to BDE-47-activated AHR caused autophagy dysfunction and led to alterations in the arrangement of HCs and supporting cells, OHC loss and elevated ABR thresholds [78]. Table 2 summarizes the results obtained from studies that have correlated ototoxicity to autophagy status.

## 6. Autophagy and Noise-Induced Hearing Loss

It has been shown that autophagy can protect the auditory system from the increase in ROS levels caused by exposure to high levels of noise [118]. Importantly, autophagy induction is dependent on the degree of oxidative stress, which is, in turn, determined by the intensity of the noise signal. Studies conducted by Yuan and co-workers (2015) [118] demonstrated that autophagy was sharply induced in CBA/J mice after temporary threshold shifts (TTS) but it rose only slightly in response to permanent threshold shifts (PTS) and it was not induced in response to severe PTS (sPTS). Therefore, lower levels of oxidative stress induce a protective response against noise-induced HL (NIHL), promoting OHC survival, but excessive oxidative stress overwhelms the beneficial potential of autophagy in OHCs and leads to OHC death and NIHL. Rapamycin administration to mice that presented TTS decreased the levels of oxidative stress and reduced noise-induced OHC loss; the opposite effect was observed when autophagy was inhibited with 3-MA [118]. Increased autophagic activity in NIHL mice has also been described by Ye and co-workers (2019) [23], with augmented levels of acetylated HSP70 protein promoting the formation of autophagosomes and a decrease in oxidative stress in OHCs.

Some of the compounds that offer protection against NIHL act through the activation of autophagy. Exposure to moderate levels of noise induced an up-regulation in the expression of the Ca^2+^/calmodulin-dependent protein phosphatase calcineurin in OHCs [119], due to a sustained elevation of intracellular Ca^2+^ levels. Calcineurin activation led to dephosphorylation of nuclear factor of activated T-cells isoform c4 (NFATc4/NFAT3) in OHCs and its translocation from the cytoplasm to the nucleus; increased nuclear NFAT3 induced OHC death. These changes were attenuated by treatment with the immunosuppressant FK506 (tacrolimus), which ameliorated increased NFAT3 nuclear translocation, noise-induced ROS production, HC loss and HL. FK506 bound to the FK506-binding protein FKBP12 which bound to calcineurin, inhibiting it. It behaved as a stronger autophagy inducer than rapamycin and its mechanism of protection was partly mediated by an elevation in LC3B expression in OHCs, since silencing of the latter partially abolished the protection conferred by the drug and resulted in greater OHC loss and elevated hearing thresholds [119]. Interestingly, decreased LC3B expression only affected the outcome of FK506 treatment against noise-induced damage, as LC3B levels did not change following noise exposure nor did silencing of LC3B itself have an effect against noise-induced damage [119]. Chen and co-workers (2025) [120] reported attenuated noise-induced HC loss and reduced hearing impairment in an NIHL mouse model following treatment with the lysine-specific demethylase 1 inhibitor tranylcypromine. Tranylcypromine activated autophagy through a pathway dependent on sestrin 2, a stress sensor that inhibited ROS production and down-regulated pro-inflammatory cytokine expression, halting cochlear cell apoptosis [120]. Studies by Defourny and collaborators (2019) [121] unveiled a protective effect of the peroxisome-associated protein pejvakin, a member of the gasdermin family, which modulated the recruitment of LC3B, protecting HCs against noise-induced cell damage. In another series of experiments, Jin and colleagues (2025) [35] demonstrated an induction in the expression of VDAC1 following noise exposure which resulted in ROS generation and increased TNF-α (tumor necrosis factor-α) levels. Blockade of VDAC1 reduced oxidative stress and protected HC synaptic ribbons through a PINK1/PARKIN-dependent pathway; it promoted mitophagy and prevented the increase in hearing thresholds induced by noise exposure [122].

Noise exposure can lead to the degradation of synapses between IHCs and SGNs, resulting in increased auditory thresholds despite IHC survival [122]; it is thought that noise-induced synaptopathy arises from excessive glutamate release from the IHCs coupled to a decreased ability for glutamate uptake [123] and that it is mainly mediated by Ca^2+^-permeable AMPA receptors that lack the GluA2 subunit [124]. Increased glutamate levels result in excitotoxicity and neuronal degeneration. Wang and collaborators (2025) [125] reported protection by BDNF (brain-derived neurotrophic factor) against ribbon synapse degeneration in noise exposure models. Although autophagy induction and disruption of mTOR function leads to reduced ribbon synapses in postnatal mice but has no effect on adult mice [25], these researchers demonstrated that BDNF conferred protection against synaptopathy by activating the PI3K/AKT/mTOR signaling pathway and blocking autophagy. Conversely, mTORC1 inactivation preserved ribbon synapse numbers and alleviated ARHL in aging Raptor-KO mice, compared to wild-type controls [64].

There is currently an increasing number of studies trying to identify the differential gene expression profiles in NIHL. Characterization of protein expression changes in the cochleae of NIHL mice established a key role for the activation of inflammation and autophagy pathways in NIHL [126]. A significant decrease in the expression of genes like *Fgf*1, *Akt*2 and *Atg*5 was recorded, while the expression levels of other genes like *Itga*1, *Kng*1 and *Cf*1 were up-regulated. Plasma metabolomics analyses of a group of 62 NIHL patients and another group of 62 control subjects identified a series of metabolic biomarkers associated with NIHL [127]. Two hundred and seven metabolites demonstrated differential regulation; among them, the researchers identified autophagy-related genes like *Pi3k*, *I3k*, *Akt*, phosphatidylethanolamine and *Atg*5 as being significantly down-regulated in NIHL patients and a close relation was established between autophagy and the development of NIHL. Using an NIHL mouse model that presented OHC loss and elevated ABR thresholds, Miao and co-workers (2022) [128] identified nine differentially regulated metabolic pathways and 17 differential metabolites. The autophagy inducer spermidine was the most differentially regulated metabolite, with augmented levels in noise-exposed animals. Spermidine was associated with an increase in the levels of LC3B and Beclin 1 in SGNs of NIHL animals, pointing to these proteins as important molecular regulators during the response to noise stimulation.

Single nucleotide polymorphisms (SNPs) in genes related to metabolic pathways such as oxidative stress, apoptosis and potassium recycling have been closely associated with NIHL. Miao and collaborators (2025) [129] conducted genotyping for six SNPs located in the genes *Atg*4c, *Atg*5 and *Atg*7 in a study that included 688 patients suffering from NIHL and 667 individuals with normal hearing. They identified a significant correlation between the genotypic distribution of *Atg*5 rs510432 and hearing status. The presence of a rs510432 CT/TT genotype instead of a CC genotype correlated with a greater expression of *Atg*5, higher levels of the antioxidant enzymes SOD and GSH-Px and lower caspase-3 levels, together with reduced susceptibility to NIHL. The group located the rs510432 sequence close to a binding site for C/EBPb in the *Atg*5 promoter; the rs510432 CT/TT sequence altered the binding efficiency of C/EBPb to the *Atg*5 promoter, leading to an increased transcription of the gene and the promotion of autophagy. Table 3 summarizes the data obtained from studies that have correlated NIHL with autophagy status.

## 7. Autophagy and Mutation-Induced Hearing Loss

Mutations in as many as 156 genes have been identified to date as causing non-syndromic SNHL (https://hereditaryhearingloss.org/ (5 January 2026), updated on 19 February 2025) and it is estimated that hereditary factors account for approximately 60% of SNHL cases and affect about 1 out of every 500 neonates worldwide [130,131]. Some of the mutations affect genes involved in the autophagic process [132,133]. Additionally, some other mutations may give rise to proteinopathies, whereby abnormal protein aggregates accumulate in the inner ear, disrupting the autophagy–lysosomal pathway [134]; an example of these are mutations in the oxysterol binding protein-like 2 (*OSBPL*2) gene, which cause hearing impairment that is attenuated by rapamycin treatment [133]. Another example are mutations in the *KCNQ*4 gene, which encodes the voltage-dependent potassium ion channel Kv7.4, leading to non-syndromic SNHL. The channel is abundantly expressed in OHCs and the accumulation of truncated mutant proteins lacking the C-terminal domain induced ER stress and ultimate cell death. Application of the autophagy inducers imatinib mesylate, SB202190 and FK-506 ameliorated cytotoxicity, while autophagy inhibition by chloroquine aggravated it [135]. A role for the lysosome–autophagy pathway has been put forward for hereditary sensory and autonomic neuropathy type 1E (HSAN1E), a genetic neurological disorder associated with mutations in the *DNMT*1 (DNA methyltransferase) gene [136]. HL is the most frequent symptom in these patients and it has been linked to the formation of aggresomes in the cytosol, inclusion bodies for the disposal of misfolded mutant DNMT1 protein through the autophagic pathway; accumulated aggresomes give rise to cellular stress that especially affects the sensory and autonomic neurons of the peripheral nervous system. A deficiency in the NPC intracellular cholesterol transporter 1 (NPC1) in Niemann-Pick type C disease (NPCD) leads to cholesterol accumulation and increased expression of the nuclear receptor coactivator 4 (NCOA4), which promotes iron release. Liang and colleagues (2022) [137] reported that NPC1 deficiency promoted autophagy-dependent ferritinophagy and ferroptosis, with subsequent HC loss and high-frequency HL; the application of autophagy inhibitors (e.g., chloroquine, wortmannin) reduced NCOA4 expression and iron levels.

Other alterations in the lysosomal pathway are found in patients with mutations in the *ATP*6V1B1 gene that encodes the B1 subunit of the V-ATPase proton pump, who develops early-onset SNHL. *Atp*6v1ba-deficient zebrafish exhibit HC degeneration that does not run parallel to changes in the levels of cleaved caspase-3 but to an imbalance in lysosomal pH and autophagic impairment [138]. There are other disorders presenting hearing impairment (e.g., Gaucher disease, Fabry disease, Pompe disease) that are associated with lysosomal alterations resulting from mutations in genes encoding proteolytic enzymes in the lysosomes; these mutations are likely to result in an altered pH within the lysosome and impaired degradation of the autophagosomal contents, and thus in autophagy dysfunction [23].

Progressive SNHL has been recorded in patients carrying mutations in the cationic amino acid transporter *SLC*7A14 (solute carrier family 7 member 14) gene. SLC7A14 is localized to intracellular membranes and mediates lysosomal uptake of arginine; it is markedly up-regulated in IHCs, compared to OHCs, and deletion or mutation of the gene leads to aberrant lysosomal homeostasis, disruption of autophagy and progressive IHC degeneration in adult mice, without affecting OHC function [139]. Similarly, mutations or deletion of the *SLC*4A11 gene are linked to HL. The protein localizes to the inner mitochondrial membrane and its expression is induced by oxidative stress by the transcription factor NRF2. *SLC*4A11 mutations trigger dysfunctional autophagy during mitochondrial oxidative stress [140].

Mutations in the PDZ domain-containing protein GIPC3, which belongs to the GAIP interacting protein C-terminal gene family, trigger hereditary non-syndromic deafness [141]. GIPC3 knock-down in HEI-OC1 cells resulted in a reduction in the levels of the early endosomal marker APPL1, impairing early endosome-dependent mitophagy and promoting ROS generation and accumulation of cytoplasmic mitochondrial DNA [142]. GIPC3 knock-down ultimately led to a reduction in the viability and proliferation of the cultures and altered mitochondrial metabolism, exacerbating the response to oxidative stress conditions. The work carried out by Li and co-workers (2025) [142] thus revealed a key role of this protein in early endosome-dependent mitophagy. GIPC3 also interacts with DRP1 [143], directing its aggregation in perinuclear mitochondria and promoting mitochondrial fission and mitophagy. Non-syndromic deafness has also been associated with mutation in the *CIB*2 (calcium- and integrin-binding protein 2) gene [144]. CIB2 binds to the inactive form of the mTORC1 activator RHEB and maintains it inactivated; thus, reduced CIB2 levels result in mTORC1 hyperactivation.

Additionally, SNHL may be a component of complex clinical syndromes associated with specific gene mutations. An example is the Warsaw breakage syndrome, where mutations in the DNA helicase DDX11, which is involved in DNA repair, lead to defects in autophagosome biogenesis [145]; these defects are linked to an impairment of the ATG16L1 precursor trafficking and maturation, to decreased LC3 lipidation, and thus to reduced autophagic flux.

Some mitochondrial mutations have been linked to hearing impairment. The 12S ribosomal RNA 1555A>G mutation leads to impaired synthesis of mitochondrial-encoded proteins and respiratory deficiencies. This in turn results in abnormal mitochondrial morphology, decreased mitochondrial membrane potential and increased production of reactive oxygen species, together with an up-regulation of autophagy, mitophagy and apoptotic pathways [92,146]. Mutations in miR-96 have been associated with HL [147]. Reduced miR-96 expression up-regulated the level of ATG7, which led to increased synthesis of autophagosomes, hyperactivation of the autophagic flux and neuronal degeneration [148].

Studies on the relation between mutations in autophagy genes and SNHL have been aided by the generation of mutant animal models. Zhou and colleagues (2020) [149] recorded stereocilium damage, presynaptic ribbon degeneration and OHC loss in mice with genetic ablation of the *Atg*7 gene. The animals developed early-onset profound HL resulting from impaired autophagy and mitophagy and an accumulation of dysfunctional mitochondria. Mutations in the *Gjb*2 gene, which encodes the connexin 26 protein, caused oxidative stress and impaired autophagy, leading to accelerated HC degeneration [150]. *Gjb*2 knock-out mice exhibited increased apoptosis and a down-regulation in autophagic activity in Kölliker’s organ at P1 [151]. The mutation caused a reduction in gap junction coupling, leading to a drop in glucose levels that could not be overcome due to the reduced autophagic activity; the concomitant reduction in ATP levels ultimately affected ATP-dependent Ca^2+^ signaling in Kölliker’s organ that was crucial for the correct development of the auditory system. Mutant animal models have also been used to carry out transcriptomic analyses, as reported by Liu and co-workers (2021) [152]. Their group identified a whole set of differentially regulated genes when comparing normal hearing mice to animals carrying a deletion in the *Lbh* (limb-bud-and-heart) gene, which presented progressive hearing loss. Among the genes identified, they registered changes in 127 autophagy genes (e.g., *Foxo*1, *Foxo*3, *Bcl*2) in mutant OHCs.

Hearing loss has also been associated with genetic factors other than mutations. Using a thioredoxin (*Trx*) transgenic mouse model, Ren and colleagues (2021) [153] established a link between diabetes and hearing impairment. The antioxidant TRX has been reported to be down-regulated in diabetes and the mice presented diabetes-induced HC degeneration which was reduced when TRX was overexpressed. TRX expression increased mitochondrial biogenesis and decreased the mitochondrial apoptotic pathway; LC3-II levels were up-regulated and those of P62 down-regulated, suggesting that TRX activates autophagy. Hearing impairment has also been described in APOE mice, associated with demyelination in their cochleae and an impaired clearance of the myelin debris due to altered phagocytic capacity of the cochlear macrophages and reduced autophagy [154]. Other studies have demonstrated an association between NIHL and single nucleotide polymorphisms (SNPs) of HSP70 in two large-scale gene screening programs in a noise-exposed population. One of the alleles correlated with increased levels of serum HSP70 and it was considered to promote autophagosome formation and to enhance the autophagy-mediated removal of noise-induced oxidative stress products [23]. Alternative splicing of autophagy- and mitochondria-related genes following noise exposure may also lead to differential gene expression, affecting susceptibility to NIHL [155].

## 8. Discussion

Mounting evidence supports a crucial role for autophagy in the development and homeostasis of the auditory system and demonstrates its involvement in altered sound perception. As an organ containing post-mitotic cell types which are enriched in long-lived proteins (LLPs), the cochlea must count on tightly regulated mechanisms to dispose of aberrant proteins and organelles; any disruption in these processes brings about serious consequences for the cells’ survival and affects the hearing function. Clearance of LLPs is mainly carried out through autophagy pathways, rather than through the activity of the proteasome, which mainly participates in the degradation of short-lived proteins. Additionally, disposal of dysfunctional mitochondria, crucial for the control of ROS generation and for cell survival, is carried out through the process of mitophagy, a form of autophagy. Therefore, autophagy is becoming a clear therapeutic target for the prevention of HL and it is thus not surprising that the study of the autophagic function is attracting an ever-growing interest within the field of hearing research.

Preventive regulation of autophagy to reduce the risk of developing HL could be applied in a good number of instances [156], such as when preparing to receive ototoxic drug treatment (e.g., cisplatin, aminoglycoside antibiotics) or when working in noisy environments. Nonetheless, damage to otic cell types and hearing impairment often result from alterations in multiple biological processes and the activation of different cell death pathways, making the precise regulation of autophagy during these processes far from straightforward.

Numerous reports point to the inhibition of autophagy as a key mechanism leading to HC loss and the development of hearing disorders; in these cases, induction of the autophagic flux yields positive results. However, in some other instances, hearing impairment is the outcome of excessive autophagy activation and thus suppression of this activity is demanded. Additionally, autophagy status may depend on the type and levels of the damaging agent (e.g., concentration of an ototoxin, noise intensity) and on the length and timing of its application, and therefore the effects of applying an autophagy modulator may vary [119]; there are reports on an initial cytoprotective effect followed by a cell death-inducing action of autophagy after exposure to some ototoxic agents [106]. These divergences highlight the great importance of developing biomarkers to monitor autophagy and cochlear damage prior to any therapeutic attempt. Interestingly, it has been shown that the expression profile of autophagy-related genes varies according to the degree of HL [49].

New technological advances are now available that allow the identification of non-invasive biomarkers, easily obtained from biological samples such as blood, saliva, and urine [157]. These biomarkers can be subjected to frequent testing and permit a dynamic analysis of transcriptional regulation, epigenetic changes, proteomics and metabolic shifts associated with damage to the cochlear cell types and progression to HL [127,128,155]. Significant changes have been identified in various miRNAs following cochlear damage that run parallel to alterations in autophagy [69,70,71,72,98,99]; of note, miRNA-based biomarkers in the blood have already shown their value as indicators of aminoglycoside antibiotic-induced ototoxicity [158].

Epigenetic changes have been described that depend on the concentration of ototoxin that the subject is exposed to; Mu and colleagues (2023) [98] registered an induction of H3K9me2 that led to the down-regulation of FOXG1-mediated autophagy and increased HC death following exposure to high cisplatin concentrations. Importantly, factors other than the damaging agent and the exposure regime modulate the autophagic response. These range from external factors, such as chronic sleep deprivation, which has been associated with the disruption of autophagy and a greater susceptibility to auditory damage [159], to genetic variations. Differences in the expression of autophagy-related genes have been identified in NIHL patients that were due to alternative splicing events following noise exposure, thus establishing a link between these events and susceptibility to NIHL [155]. Also, SNPs have been identified in the HSP70 gene in noise-exposed individuals, with one of the alleles correlating with increased levels of serum HSP70, increased autophagic activity and diminished ROS accumulation [23]. Similarly, the presence of the *Atg*5 rs510432 CT/TT genotype correlated with higher ATG5 levels, autophagy induction and reduced susceptibility to NIHL [129].

A good number of autophagy inducers act through the inhibition of the mTOR kinase, which is hyperactivated in many HL cases. mTOR is the catalytic unit of two functionally different complexes, mTORC1 and mTORC2, which have different functions in the tissues. While inhibition of mTORC1 by drugs like rapamycin leads to autophagy induction in the cochlea and appears as a promising therapeutic strategy against HL [160], inhibition of the mTORC2 pathway has resulted in severe HL. Thus, an essential role has been established for mTORC2 in the protection against HL induced by exposure to either high levels of noise or to cisplatin [161]. Hong and co-workers (2025) [162] also reported a protective effect of (-)-butaclamol against GM-induced ototoxicity, through the activation of AKT/mTOR and ERK pathways and the inhibition of the inflammatory regulator NF-kB; however, the contribution of the mTORC1 and the mTORC2 complexes was not analyzed. Therefore, further characterization of the activities of each mTOR complex in the cochlea is necessary, together with the development of isoform-specific mTOR inhibitors that are selective for their mTOR complex target. mTORC1-specific modulators have been described [163]; however, it has been seen that prolonged application or high doses of rapamycin, an mTORC1 inhibitor, also reduce mTORC2 signaling [162,164], giving rise to undesired effects such as those reported by Hong et al. (2025) [162] in the cochlea. Other drawbacks associated with the use of rapamycin are its effects as an immunosuppressant and possible systemic toxicity. Moreover, resistance to mTOR inhibitors has been reported following long-term use of these drugs; in addition, opposite effects have also been registered with these inhibitors, depending on the timing of the application. Considering these facts, as well as the participation of the mTOR signaling pathways in multiple cellular processes, other approaches have been followed consisting of the induction of autophagy through mTOR-independent mechanisms [57,165,166]; examples of this type of compound are (-)-butaclamol, which activated the AKT/mTOR pathway while restoring autophagy by reducing ROS accumulation [162], the curcumin analog C1 [47], which promoted TFEB nuclear translocation and enhanced autophagy without inhibiting mTOR, and MSL, another autophagy enhancer that activated calcineurin, promoting the dephosphorylation and translocation of TFEB to the nucleus [167].

Very importantly, autophagy dysfunction leading to ARHL has been associated with neurocognitive disease [168]. Aberrant autophagy has been linked to a collapse of the mitochondrial network, resulting in a switch from oxidative phosphorylation to glycolysis and thus in decreased ATP synthesis and reduced glucose metabolism in the hippocampus, correlating with cognitive decline.

Altogether, these data underline the relevance of characterizing the autophagy status along the processes leading to HL as well as the need to develop biological tools that allow a precise modulation of the autophagic flux in the cochlea.

## Figures and Tables

**Table 1:** Studies correlating autophagy status and cell and hearing loss in aging models.

Experimental Model	Summary of Results	Reference
ARHL rats	Decreased SIRT1 and GDF15 levels in aging rats, with high apoptosis rates and blocked autophagy GDF15 overexpression restores autophagic flux and improves ABRs via SIRT1 up-regulation	[16]
C57BL/6J mice, aged HEI-OC1 cell cultures (H_2_O_2_)	HC senescence and autophagy are up-regulated in aged mice PIN1 down-regulation induces autophagy and exacerbates HL Autophagy inhibition reduces senescence and aging	[46]
CBA/J mice	Possible epigenetic silencing of HC genes and down-regulation of autophagy and mTOR signaling during aging	[48]
Mouse microarray data	Different sets of differentially expressed autophagy genes depending on degree of HL	[49]
C57BL/6J mice	Oxidative stress, increased SGN apoptosis, increased mTOR activity and decreased expression of *Atg* genes and autophagosomes in aging mice Rapamycin increases autophagy, improves ABRs, reduces SGN apoptosis and enhances SGN density	[50]
C57BL/6, aged HEI-OC1 cells and cochlear explant cultures (D-galactose)	C1 exerts an antioxidative effect, abolishes aging-associated inhibition of TFEB nuclear translocation, alleviates senescence, reduces HC loss and improves ABRs in aged mice	[47,56,57]
C57BL/6J mice	Antioxidant thymoquinone down-regulates BAK1 and up-regulates the autophagy inducer SIRT1 Thymoquinone preserves HC morphology and enhances SGN survival in aging mice, delaying ARHL onset	[58]
C57BL/6J mice, aged HEI-OC1 cell cultures (H_2_O_2_)	Enhanced expression of PINK1 and Parkin in HCs of aged mice and delay in ARHL progression following SIRT1 overexpression	[59]
SAMP8 mice	Autophagic stress: - Initial pro-survival role of autophagy - Excessive autophagy activation in older animals, damaged mitochondrial and protein aggregates, SGN death	[61,62]
C57BL/6J mice, HEI-OC1 cell cultures	Up-regulation of HDAC11 in HCs of aging mice Inhibition of PINK1 and Parkin by HDAC11, with impaired mitophagy and mitochondrial dysfunction and attenuation of ARHL	[62]
C57BL/6J, HEI-OC1 cell cultures	Dysregulated mTORC1 activity during aging, contributing to ARHL mTORC1 inhibition in aged animals promotes HC survival and preserves synaptic ribbon numbers Constitutive mTORC1 activation leads to autophagy inhibition, ROS-induced premature senescence, accelerated HC loss and early-onset progressive HL	[64,65]
Aged HEI-OC1 cell cultures (NaAsO_2_)	Premature senescence results from high ROS accumulation, TFEB inactivation and blockade of autophagosome–lysosome fusion mTOR inhibition promotes autophagy and reduces % β-galactosidase-positive cells	[66]
Cochleae from aged FVB mice, aged HEI-OC1 cells and cochlear explant cultures (D-galactose)	Low D-galactose concentrations induce autophagy; higher concentrations disrupt normal autophagy, induce mitochondrial damage and HC senescence Overexpression of RONIN increases autophagy and lysosomal activity, increases TEFB activity and leads to enhanced HC survival	[67]
C57BL/6J mice, aged cochlear explant cultures (D-galactose)	Neuregulin overexpression in supporting cells increases autophagy and decreases caspase-3 activity, attenuating D-gal-induced senescence of HCs and SGNs and preserving hearing	[68]
C57BL/6J mice, aged HEI-OC1 cell cultures (D-galactose)	Excessive autophagy and up-regulation of the DLK/JNK pathway are associated with senescence Inhibition of autophagy, DLK or JNK3 reduces senescence and improves hearing	[69]
GFP-LC3B mice, OC-1 cell and cochlear explant cultures (D-galactose)	Decreased FOXG1 levels in aged cultures, further reduced by inflammatory damage FOXG1 behaves as an autophagy regulator and exerts a protective role against inflammatory injury Extensive cell damage induces a reduction in FOXG1 levels and increases apoptotic rates	[70]
C57BL/6 mice, HEI-OC1 cell cultures	Up-regulation of miR-34a correlates with down-regulation of the ATG9A protein and an impairment of the autophagic flux, HC loss and ARHL miR-34a suppression restores autophagy and protects cells from death	[71]
C57BL/6 mice, HEI-OC1 cell cultures	Increased levels of miR-130b-3p and reduced expression of PPARγ in the cochleae of aged mice that present hearing impairment miR-130b-3p down-regulation leads to increased expression of PPARγ, ATG5, Beclin 1 and LC3B-II	[72]
C57BL/6 mice, HEI-OC1 cell cultures	Rapamycin treatment restores autophagy in aging HCs, preserves HC numbers and delays ARHL	[73]
CBA/J mice, HEI-OC1 cell cultures	Cholesterol accumulation leads to increased mTORC1 signaling and reduced TFEB levels Treatment with atorvastatin preserves lysosomal function and autophagy by inhibiting mTORC1 and activating TFEB, resulting in delayed HL	[74]

**Table 2:** Studies correlating autophagy status and ototoxicity.

Experimental Model	Summary of Results	Reference
Wistar rats	Attenuation of cisplatin-induced HL through autophagy activation, which reduces oxidative stress, auditory threshold shifts and OHC loss	[32]
C57BL/6J mice, cochlear explant cultures	Rapamycin counteracts cisplatin-mediated activation of mTORC1 leading to ROS accumulation and cell death	[33]
Guinea pigs, HEI-OC1 cell cultures	Increased intracellular ROS levels, autophagy inhibition, OHC loss and elevated ABR thresholds following treatment with the pollutant BDE-47, which induces AHR expression	[78]
Various knock-out mouse lines, HEI-OC1 cell cultures	Translocation of RIPOR2 upon AG entry into HCs leads to recruitment of autophagy-related proteins and GABARAP, autophagy dysregulation, HC death and HL	[80,81,82]
C57/BL6J mice, cochlear explant cultures	GM induces mTORC1 hyperactivation and leads to decreased SGN densities and reduced neurite outgrowth	[83]
HEI-OC1 cell cultures, zebrafish larvae	Osthole restores autophagy and attenuates oxidative stress and HC apoptosis in HEI-OC1 cells and zebrafish larvae treated with GM	[84]
Sprague-Dawley rats, HEI-OC1 cells, cochlear explant cultures	GM treatment causes HL and induces the accumulation of LC3 GM causes delayed ototoxicity in cochlear explants and HEI-OC1 cultures, with a time-dependent increase in LC3-II and Beclin 1 expression and decreased RAB7, autophagosome accumulation and cell death	[85]
Cybrid cell lines generated by transferring mitochondria from immortalized lymphoblastoid cell lines into human cells lacking mitochondrial DNA	GM aggravates mitochondrial dysfunction in cybrids carrying a m.1494C>T mutation in mitochondrial rRNA. Strong stimulation of mitophagy to eliminate damaged mitochondria and protect cells from apoptosis	[86]
Murine cochlear explants	Increased autophagy induction in GM-treated murine cochlear cultures Autophagy inhibition protects against HC loss; interference of 3-MA with the pro-apoptotic function of ATG12	[87]
HEI-OC1 cell cultures	Autophagy induction in neomycin-treated HEI-OC1 cultures, with inhibition of miR-489 and increased NDP52 levels Fasudil-mediated inhibition of neomycin-induced ROS production and mitophagy activation and protection against apoptosis	[88]
C57BL/6 mice, HEI-OC1 cells, cochlear explant cultures	Umbilical MSC-derived exosomes activate autophagy and promote the survival of HEI-OC1 and cochlear explant cultures Requirement of endocytosis for autophagy activation	[89]
Guinea pigs, Sprague-Dawley rats, HEI-OC1 cell cultures	Inhibition of PDE4 promotes autophagy in kanamycin-treated models and preserves cell viability, IHC ribbon synapses and hearing function	[90]
C57BL/6J mice	Kanamycin treatment suppresses autophagosome formation mTOR inhibition by temsirolimus promotes TFEB nuclear translocation and autophagy and attenuates SGN degeneration, reverting the effects of kanamyicn	[91,92]
C57BL/6 mice	Promotion of apoptosis and autophagy in mice with cisplatin-induced HL	[94]
C57BL/6 mice	Increased autophagy as a protective response in cisplatin-treated SGNs, mediated by PRDX1 No effect of PRDX1 expression on HC survival following cisplatin treatment	[95]
C57BL/6 mice, HEI-OC1 cell cultures	Protective effect of YTHDF1 against cisplatin-induced toxicity, by promoting the translation of ATG14 and enhancing autophagy	[96]
C57BL/6 mice, HEI-OC1 cells, cochlear explant cultures	Activation of the PINK1/Parkin pathway by cisplatin, enhancing autophagy and inhibiting the p-JNK apoptotic pathway PINK1 silencing leads to decreased autophagy and more severe cisplatin-induced apoptosis	[97]
CBA/CaJ mice, OC-1 cell cultures	Increased FOXG1 expression under low concentrations of cisplatin Increased H3K9me2, reduced FOXG1 levels and autophagy, increased ROS and HC death following treatment with high concentrations of cisplatin, which cause a reduction in the expression of miR-34a, miR-96, miR-182 and miR-183	[98]
C57BL/6 mice, HEI-OC1 cell cultures	Increased miR-34a expression following cisplatin treatment, with suppression of DRP1 and mitophagy inhibition Increased DRP1 expression following miR-34a inhibition leads to reduced ROS levels and attenuation of cisplatin-induced ototoxicity	[99]
C57BL/6J mice, HEI-OC1 cells, cochlear explant cultures	Trehalose ameliorates cisplatin-induced oxidative stress, induces TFEB expression and nuclear translocation and reduces HC loss Cyclosporin A prevents TFEB nuclear translocation and promotes cisplatin-induced apoptosis	[100]
C57BL/6 mice, zebrafish, HEI-OC1 cell cultures	Metformin induces autophagy and increases cell survival of cisplatin-treated HEI-OC1 cultures Metformin preserves HCs in zebrafish and mice treated with cisplatin Metformin likely acts through AMPK/FOXO3-mediated autophagy	[101]
HEI-OC1 cell cultures	Involvement of AMPK in the protective action of metformin AMPK inhibition abolishes the effects of metformin on HEI-OC1 cells treated with the oxidative stress inducer tert-butyl hydroperoxide	[102]
C57BL/6 mice, HEI-OC1 cell cultures	Activation of autophagy by a chiisanoside derivative that up-regulates LRP6 and inhibits the negative regulator of autophagy GSK3β	[103]
Sprague-Dawley rats, HEI-OC1 cell cultures	Inhibition of GSK3β promotes nuclear translocation of TEFB and inhibition of ROS generation	[104]
C57BL/6 mice, HEI-OC1 cell cultures	Nobiletin attenuates apoptosis and oxidative stress through autophagy activation, with nuclear translocation of NRF2	[105]
HEI-OC1 cell cultures	Cell death-promoting effect of autophagy following an initial cytoprotective effect against cisplatin treatment Autophagy inhibition confers protection against cisplatin	[106]
HEI-OC1 cell cultures	Meclofenamic acid inhibits excessive autophagy activation by cisplatin and reduces oxidative stress and apoptosis	[107]
Zebrafish larvae, HEI-OC1 cell cultures	Lysophosphatidic acid protects against cisplatin-induced HC death by reducing oxidative stress, autophagy and apoptosis	[108]
C57BL/6-129/SvEv mice, cochlear explant cultures	Induction of STAT1 by cisplatin and GM STAT1 knock-down induces autophagy and protects HCs from ototoxicity	[111]
C57BL/6J mice, HEI-OC1 cells, cochlear explant cultures	Paracetamol may cause ototoxicity by decreasing the activity of lysosomal enzymes and disrupting autophagy	[112]
C57BL/6J mice, cochlear explant culture	Sevoflurane can cause HL by reducing cochlear ribbon synapses and decreasing autophagy	[113]
C57BL/6 mice, HEI-OC1 cell cultures	Ototoxicity associated with high doses of caffeine due to activation of autophagy and apoptosis via the SGK1/HIF-α pathway	[114]
C57BL/6J mice, BALB/c mice, HEI-OC1 cells, cochlear explant cultures	Ototoxicity associated with elevated levels of uric acid, through ferritinophagy Autophagy blockade ameliorates uric acid-induced cytotoxicity	[115]
SGN cultures	Cadmium induces SGN degeneration and HL Cadmium causes an impairment of the autophagic flux and metformin protects cultures through autophagy restoration	[116]
Guinea pigs	Lead exposure causes or aggravates HL Lead exposure leads to a reduction in the numbers of SGNs, with no effect on HCs Possible protective role of autophagy in the early stages following lead exposure	[117]

**Table 3:** Studies correlating autophagy status and noise-induced hearing loss.

Experimental Model	Summary of Results	Reference
C57BL/6, HEI-OC1 cells, cochlear explant cultures	Induction of VDAC1 following noise exposure, leading to ROS generation and increased TNF-α levels VDAC1 blockade protects HC synaptic ribbons through a PINK1/Parkin-dependent pathway, induces mitophagy and prevents the elevation of hearing thresholds induced by noise exposure	[35]
CBA/J mice, C57BL6 mice	Autophagy-mediated protection against noise-induced ROS accumulation Autophagy induction depends on the degree of oxidative stress. Strong induction of autophagy after TTS, weak induction after PTS, no induction after sPTS Rapamycin decreases levels of oxidative stress and reduces noise-induced OHC loss	[118]
CBA/J mice, HEI-OC1 cell cultures	Up-regulation of calcineurin and NFAT3 following noise exposure, which is attenuated by FK506 FK506 ameliorates ROS generation, HC loss and HL, partly through an elevation of LC3B expression in OHCs	[119]
C57BL/6J mice	Attenuated noise-induced damage by tranylcypromine, which activates autophagy through a sestrin 2-dependent pathway	[120]
C57BL/6-129/Sv mice, HepG2 and HeLa cell cultures	The peroxisome-associated protein pejvakin modulates the recruitment of LC3B and protects HCs against noise-induced cell damage	[121]
C57BL/6J mice, cochlear explant cultures	BDNF reduces ABR thresholds in noise-exposed animals and mitigates noise-induced ribbon synapse loss Protection by BDNF against synaptopathy in a noise exposure cochlear explant model. Protection conferred by the activation of PI3K/AKT/mTOR signaling, correlating with reduced autophagosome formation and lysosomal degradation	[125]
C57BL/6J mice	Key role for the activation of inflammation and autophagy pathways in NIHL	[126]
Normal hearing and NIHL subjects from within a group of workers exposed to occupational noise	Identification of metabolic biomarkers associated with NIHL, including autophagy-related genes	[127]
C57BL/6 mice	Differentially regulated metabolic pathways and metabolites in an NIHL mouse model, including the autophagy inducer spermidine, the expression of which is increased in noise-exposed animals Spermidine is associated with increased LC3B and Beclin 1 levels in SGNs of NIHL animals	[128]
Normal hearing and NIHL subjects from within a group of workers exposed to occupational noise	Significant correlation between the *Atg*5 rs510432 CT/TT genotype, a greater expression of *Atg*5, autophagy induction and reduced susceptibility to NIHL	[129]

## Data Availability

No new data were created or analyzed in this study. Data sharing is not applicable to this article.

## Abbreviations

The following abbreviations are used in this manuscript:

ABR	Auditory brainstem response
AIF	Apoptosis-inducing factor
AMPK	Adenosine monophaosphate-activated protein kinase
ARHL	Age-related hearing loss
ATG	Autophagy-related protein
BAK1	Bcl-2 antagonist/killer 1
DRP1	Dynamin-related protein 1
FOXG1	Forkhead box G1
HC	Hair cell
HL	Hearing loss
HO-1	Heme oxygenase-1
IHC	Inner hair cell
KEAP1	Kelch-like ECH-associated protein 1
LC3	Microtubule-associated protein light chain 3
LLPs	Long-lived proteins
3-MA	3-methyladenine
MMP	Mitochondrial membrane potential
mTOR	Mammalian target of rapamycin
NIHL	Noise-induced hearing loss
NQO-1	NAD(P)H:quinone oxidoreductase-1
NRF2	Nucleus factor erythroid 2-related factor 2
OHC	Outer hair cell
OMM	Outer mitochondrial membrane
PAR	PolyADP-ribose
PARP-1	Poly(ADP-ribose) polymerase-1
PINK1	Phosphatase and tensin homolog-induced kinase 1
ROS	Reactive oxygen species
SGN	Spiral ganglion neuron
SIRT1	Sirtuin 1
TFEB	Transcription factor EB
THSG	2,3,4′,5-tetrahydroxystilbene-2-O-b-D-glucoside
TSC	Tuberous sclerosis complex
VDAC1	Voltage-dependent anion channel 1
